# Case report: Early acute myocarditis after radiation therapy for breast cancer: A case presentation and review of literature

**DOI:** 10.3389/fcvm.2023.1020082

**Published:** 2023-04-19

**Authors:** Mohammadbagher Sharifkazemi, Mahsa Elahi, Masoud Sayad

**Affiliations:** ^1^ Department of Cardiology, Nemazee Hospital, Shiraz University of Medical Sciences, Shiraz, Iran; ^2^ Radiation Oncology Department, Nemazee Hospital, Shiraz University of Medical Sciences, Shiraz, Iran; ^3^ Cardio-Oncology Department, Rajaie Heart Hospital, Iran University of Medical Sciences, Tehran, Iran

**Keywords:** radiation-induced, cardiomyopathies, acute myocarditis, myocarditis, breast cancer

## Abstract

Breast cancer is the most commonly diagnosed cancer in women worldwide, and with the increased survival of patients by novel treatments, the frequency of complications of cancer treatments rises. Radiotherapy, especially on the chest wall, can damage different cardiac structures. Radiotherapy-induced cardiomyopathy mainly occurs over 10 years after breast cancer treatment; however, there is a gap in the literature on acute myocarditis following radiotherapy. Here, we present a 54-year-old woman who developed acute myocarditis shortly after 25 sessions of radiotherapy with 50 Gy of radiation, successfully diagnosed with the use of speckle tracking echocardiography (STE) and cardiac magnetic resonance (CMR), and responded to the medical treatment with relative clinical improvement until the final follow-up. This case suggests the necessity of detailed examination of patients after radiotherapy, not only for chronic occurrence of cardiomyopathy but also for acute myocarditis. Although STE and CMR resulted in accurate diagnosis, in this case, further studies are required to determine the diagnostic accuracy of these two imaging methods compared with other imaging modalities in such patients and investigate the best diagnostic tool and therapeutic approach for these patients.

## Introduction

Cardiovascular diseases (CVDs) and cancers are the top causes of death worldwide, and female breast cancer is the most diagnosed cancer with an increasing trend (1). With the advances in early diagnostic and new treatment approaches, the survival of patients improves, resulting in the increased occurrence of cancer treatment complications (2). Certain types of chemotherapy can lead to cardiovascular complications because of their cardiotoxic effects (3). Radiotherapy (RT) can also result in coronary artery disease (by the induction and acceleration of atherosclerosis through endothelial damage), as well as fibrotic damage to the valves, pericardium, and myocardium, with an estimated incidence of 10%–30%, occurring 5–10 years after treatment, known as radiation-induced heart disease (RIHD) (4).

Cardiomyopathy, induced by RT, is mainly silent (without clinical symptoms) with minor electrocardiography (ECG) changes, and the diagnosis is usually made by the abnormal findings of transthoracic echocardiography (TTE) and cardiac magnetic resonance imaging (CMR) (5). Furthermore, RT-induced cardiomyopathy generally develops over 10 years after RT, when the patient does not refer to the physician regularly, which increases the chance of missed diagnosis (6). However, the acute development of cardiomyopathy and other RIHDs should also be considered (7). Here, we present a case with the occurrence of acute myocarditis after RT for breast cancer, successfully diagnosed with the use of speckle tracking echocardiography (STE) and CMR and responded to the medical treatment until the final follow-up.

## Case presentation

A 54-year-old woman, with known case of diabetes mellitus (DM, controlled with oral hypoglycemic pills) and ischemic heart disease (left main and advanced three-vessel disease), presented to our center with complaints of dyspnea on exertion and orthopnea, with neither feverishness, sputum production, nor chest pain. She was a nonsmoker, had a body mass index of 23 kg/m^2^, her blood pressure was 105/64 mmHg, heart rate was 110 bpm, and respiratory rate was 28/min. She had no family history of cardiomyopathy. She had been on daily oral long-acting nitrate (nitrocontin 2.6 mg twice a day), 6.25 mg carvedilol thrice a day, 80 mg aspirin once a day, and 20 mg rosuvastatin once a day. Six months before her referral, left breast cancer was diagnosed for the patient (invasive ductal cell carcinoma, T1BN0M0, positive estrogen receptor), and she underwent a left-sided mastectomy (R0 resection) and conventional RT, which included 25 courses of 50-Gy external beam RT with a compact machine (6 MV photon 3D conformal therapy), delivered within 5 weeks. Computed tomography (CT) simulation was done before planning. The clinical target volume was 185.7 and the planned target volume was 548.3; we did not have gross tumor volume (GTV) because the patient underwent surgery, and the gross tumor was resected surgically. Organs at risk (OAR) included the heart, the left and right lung, and the cord. For sparing OARs, the patient was initially immobilized in the treatment position, with immobilization devices (supine breast boards); and posterior jaw was set at zero to half-beam block/beam split to minimize dose of lung and heart. Gantry angle, collimator angle, and table angle were adjusted to optimize coverage of desired targets, while minimizing normal tissue inclusion within the field. Three-dimensional (3D) treatment planning was done for delineation of the breast, heart, lungs, and the cord. All of these organs received radiotherapy less than their tolerant dose. In this plan, mean dose of the heart was 541.7 cGy. The dose–volume histogram is provided in the Supplementary Figure. After RT, the patient developed the symptoms mentioned earlier, which resulted in the referral of the patient to the physician. She remained afebrile in several visits. On physical examination, she had tachycardia and tachypnea; on auscultation, third and fourth heart sound (S3 and S4) and lung crackle were detected.

In the history, the patient was under cardiac follow-up because of the right ventricular outflow tract tachycardia (RVOT VT) with the impression of idiopathic type, left ventricular (LV) ejection fraction (EF) of 49%, and negative single-photon emission computed tomography (SPECT) for more than 2 years. Also, 24-h ECG Holter recordings showed sinus tachycardia, in addition to nonspecific ST-T changes in the precordial leads with premature ventricular complexes (PVC) of 24%, for which medical treatment with 40 mg verapamil thrice a day was prescribed 2 years ago. Four months later, with 14.7% PVC count and a drop in LV EF to 31%, the patient was scheduled for PVC ablation. However, because of the demonstration of calcified coronaries in fluoroscopy, she underwent diagnostic angiography, which determined left main and three-vessel disease, according to which the treatment strategy shifted to coronary artery bypass grafting (CABG). After the surgery, LV EF improved to 48%, and PVC count reached <1% in serial ECG Holter follow-up.

**Figure 1:**
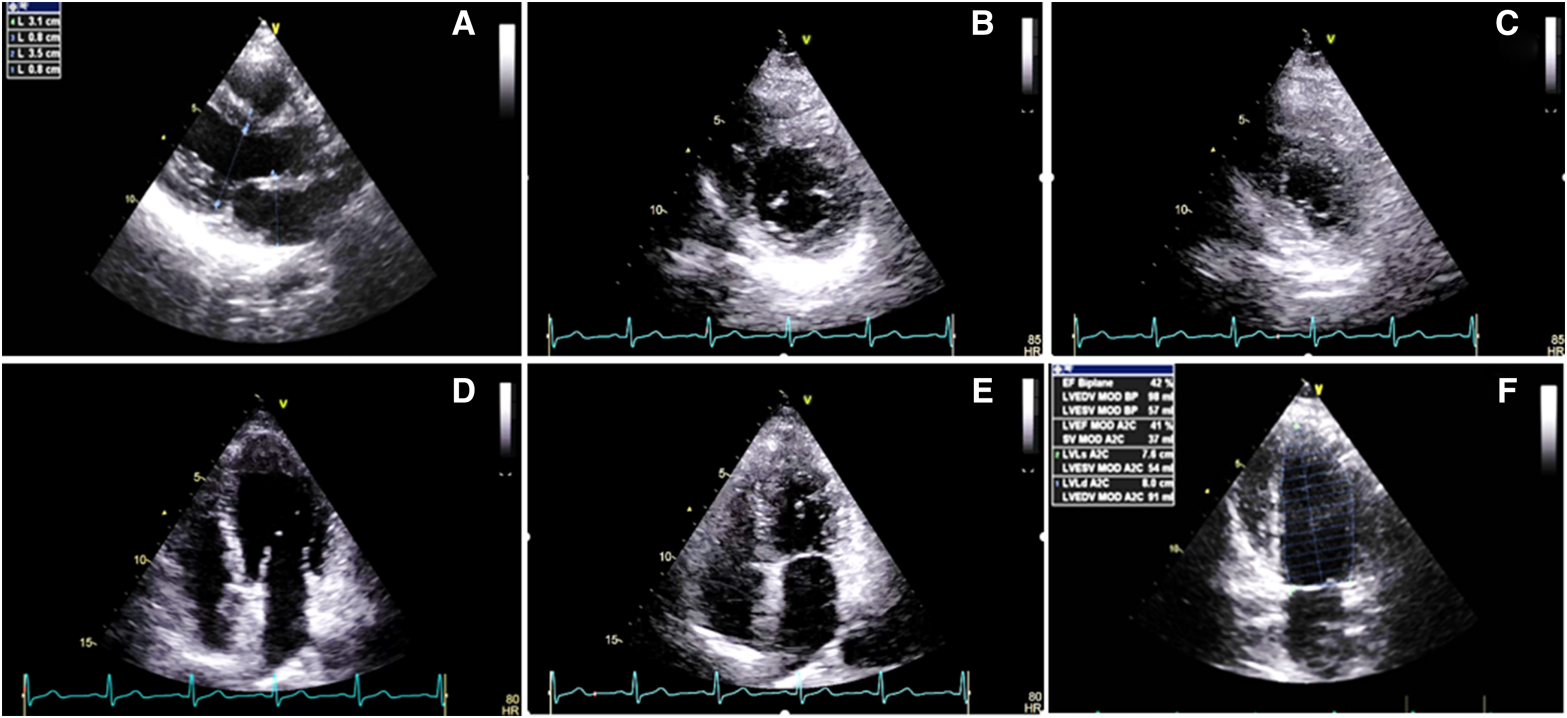
Two-dimensional transthoracic echocardiography 1 week before the radiotherapy course (**A–F**), illustrating the normal thickness of the left and right ventricular walls, in addition to global mild left ventricular systolic dysfunction: (**A**) PLAX view. (**B**) PSAX in the end-diastole. (**C**) PSAX in the end-systole. (**D**) A4C view in the end-diastole. (**E**) A4C view in the end-systole. (**F**) LV EF = 42% by Simpson's method. PLAX, parasternal long axis view; PSAX, parasternal short axis view; A4C, apical four chamber view.

**Figure 2:**
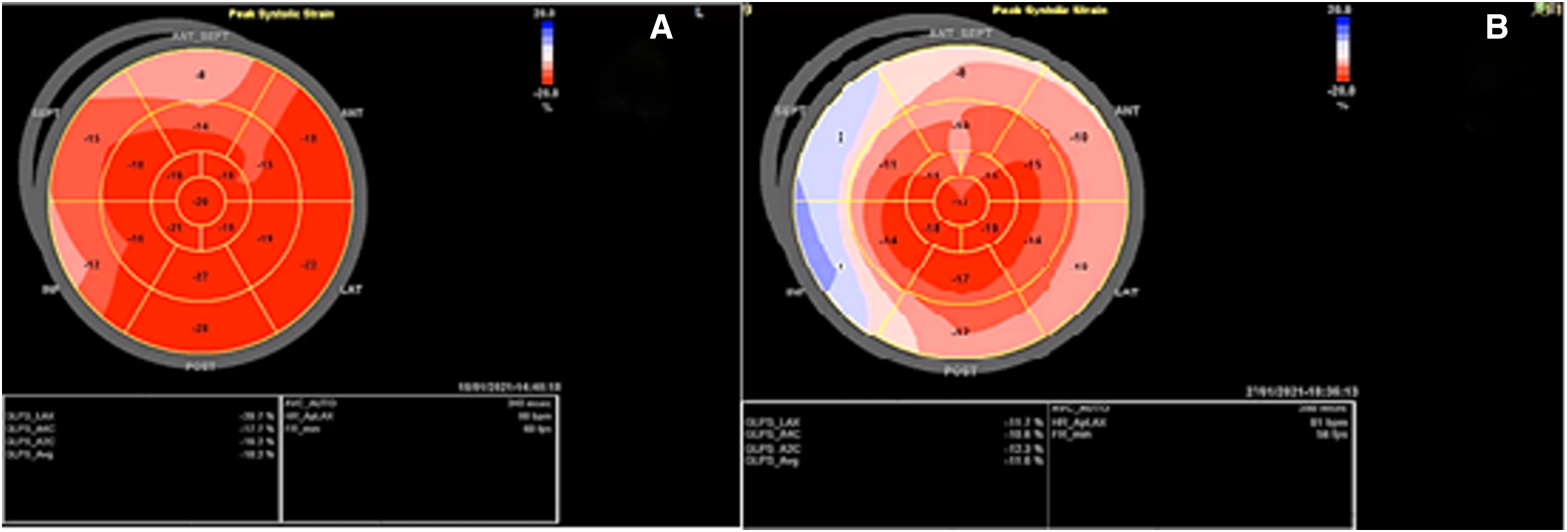
Speckle tracking echocardiography before and after the radiotherapy course: (**A**) Before radiotherapy, which showed a global longitudinal strain of −18.2% with myocardial performance impairment in the bases of inferior, septal, and anteroseptal walls a week. (**B**) After radiotherapy, which showed GLS: −11.2% with evidence of severe myocardial performance impairment accompanied by relative apical sparing.

**Figure 3:**
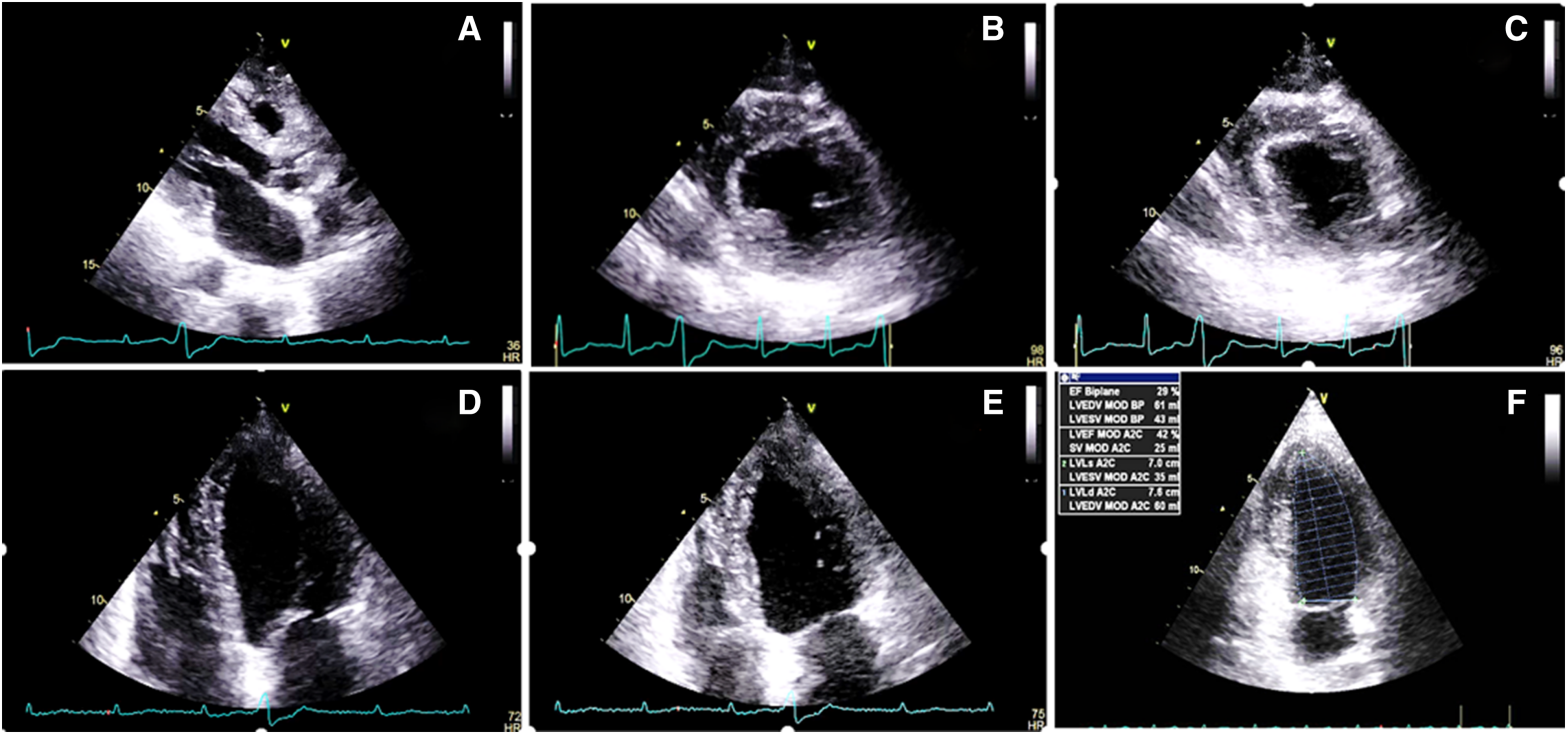
Two-dimensional transthoracic echocardiography 1 month after the radiotherapy course (**A–F**), illustrating a marked increase in the thickness of the left and right ventricular walls (15 and 7 mms, respectively), in addition to severe left ventricular systolic dysfunction. (**A**) PLAX view. (**B**) PSAX in the end-diastole. (**C**) PSAX in the end-systole. (**D**) A4C view in the end-diastole. (**E**) A4C view in the end-systole. (**F**) LV EF = 28% by Simpson's method. PLAX, parasternal long axis view; PSAX, parasternal short axis view; A4C, apical four chamber view.

Before mastectomy, TTE evaluation (performed for estimation of surgical risk) showed an LV EF of 42% and a global longitudinal strain (GLS) of −18.2% (Figures 1, 2A) with mild ischemia in the apex on SPECT. Before RT, PVC was 6% in the 24-h ECG Holter monitoring. After RT, the frequency of PVCs during 24-h Holter monitoring raised to 13.8%, accompanied by a drop of LV EF to 29% (from 42%) and GLS to −11.2% (from 18.2%, Figures 2B, 3). LV EF was measured by 2D TTE and was calculated using Simpson's method. She did not receive any course of chemotherapy at all.

The results of serum tests showed a high sensitivity troponin I level six times above the normal limit (up to 14 ng/L), evaluated using high sensitivity (hs) troponin I assay technique, high level of lactate dehydrogenase (LDH), creatine phosphokinase (CPK), aspartate aminotransferase (AST), estimated sedimentation ratio (ESR), C-reactive protein (CRP), and mild elevated total white blood cell (WBC) count (about 12,000), as well as elevated N-terminal pro-B-type brain natriuretic peptide (NT pro-BNP) (six times above the normal limit). Serum lipids were within normal limits (cholesterol = 162 mg/dl, LDL = 58 mg/dl, HDL = 41 mg/dl, and triglyceride = 166 mg/dl). The viral antibody markers were negative, which include Coxsackie virus group B, human immunodeficiency virus (HIV), cytomegalovirus, Epstein–Barr virus, hepatitis virus family, and influenza virus.

**Figure 4:**
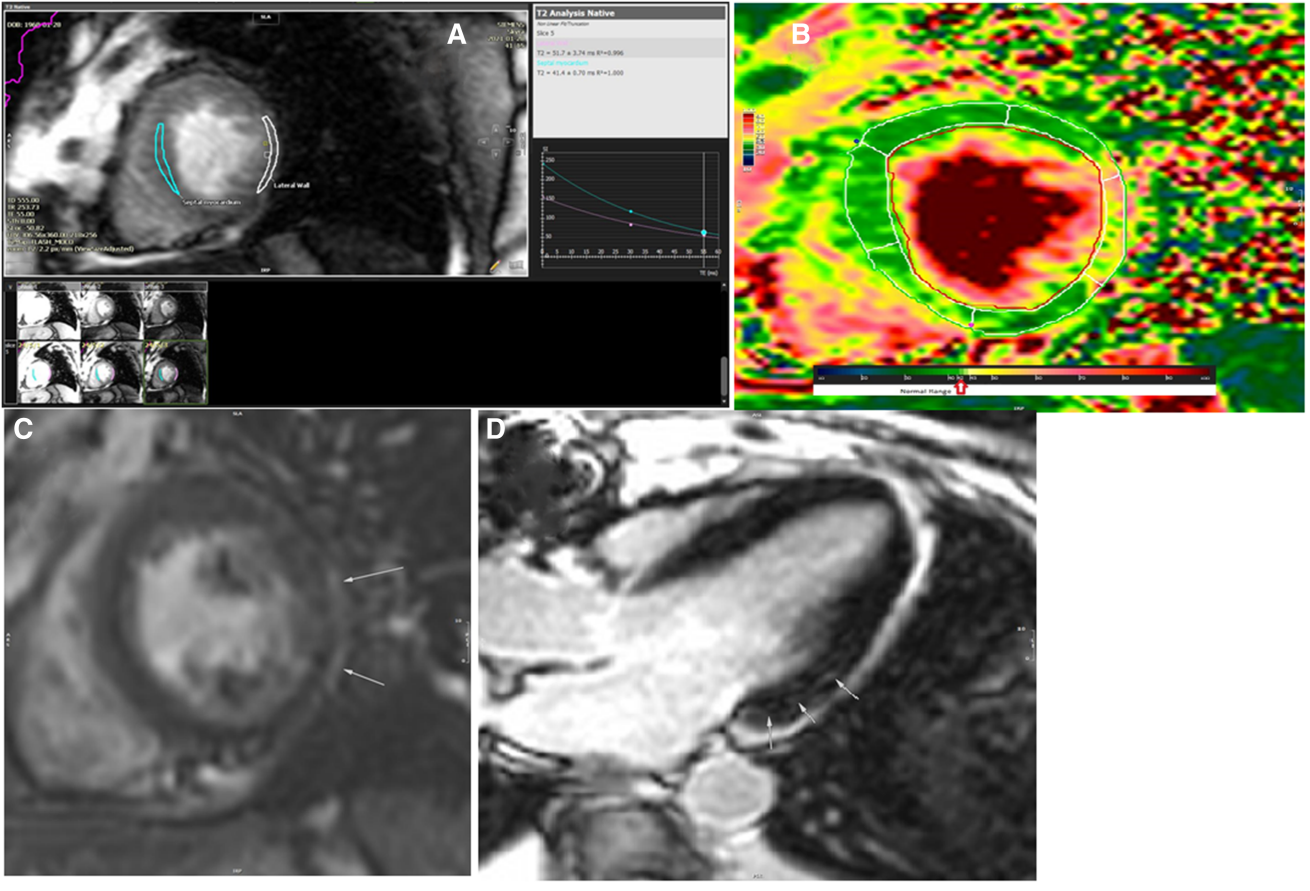
The results of cardiac magnetic resonance imaging after the radiation (**A–D**). (**A**) T2 analysis of mid-septal and mid-lateral segments of the left ventricle revealed prolongation of T2 relaxation time in the mid to apical segments of the lateral wall (52 ms). (**B**) T2 map image of the left ventricle in short axis view showed abnormal T2 value in the mid-lateral segment of the left ventricle. (**C**) Delayed postcontrast image of the left ventricle in short axis view showed a subepicardial rim of late gadolinium enhancement on the lateral wall of the left ventricle. (**D**) Delayed postcontrast image of the left ventricle in long axis three-chamber axis view revealed small focal subepicardial to mid-wall patches of late gadolinium enhancement in the basal inferolateral segment.

The patient did not accept to undergo endomyocardial biopsy. CMR was performed using a Siemens Skyra 3 T MRI scanner with the application of real-time cine images of LV, short inversion time inversion-recovery (STIR) T2-weighted image (T2WI), T2 mapping, native and postcontrast T1 mapping, and delayed postcontrast images. The results of CMR showed diffuse myocardial inflammation in T1 mapping and extracellular volume (ECV) map of LV, mid-wall late gadolinium enhancement (LGE) in basal segments of the interventricular septum (IVS), and subepicardial delayed enhancement in the basal inferolateral segments of LV without an ischemic pattern of myocardial injury (Figure 4). The regions marked as LGE on MRI refer to the presence of fibrosis in the patient. Matching the patient's clinical signs and symptoms with the European guideline for clinically suspected myocarditis, the diagnosis was considered as active myocarditis.

The prescribed medications included: carvedilol (12.5 mg twice a day), spironolactone (25 mg once a day), furosemide (40 mg twice a day), rosuvastatin (20 mg once a day), and prednisolone (10 mg once a day). Prednisolone and other medications were prescribed for improving cardiac function based on the available evidence (8, 9). Statin was prescribed considering the LDL level <60 mg/dl. At follow-up visit (2 weeks later), the patient was clinically stable without improvement of echocardiographic or STE findings: LV EF = 33% with normal LV size and focal increased myocardial thickness in the mid to apical lateral segments of the LV. Also, CT angiography at follow-up (10 months after RT) showed patent coronary grafts.

The timeline of the signs, symptoms, and diagnostic and therapeutic procedures are shown in Table 1, arranged in chronological order. On arrival, at the first cardiologist's visit (after the radiotherapy), her symptoms were classified as New York Heart Association (NYHA) class III; after the treatment, it reduced to NYHA class I. The patient gave consent for all steps of the diagnostic procedure and cooperated with the physician. The cardiologist in charge was open to listen to the patient’s opinion and reflected them in the medical records. Comparison between the values measured at the baseline and at the follow-up is provided in Table 2.

**Table 1:** Timeline of the presenting symptoms, diagnostic, and therapeutic procedures.

6 months before referral to our clinic	Coronary artery bypass graft surgery.
9 weeks before referral to our clinic	Transthoracic and STE (performed before surgery for estimation of surgical risk) showed LV EF of 42% and GLS of −18.2%, with mild ischemia in apex on single-photon emission computed tomography.
7 weeks before referral to our clinic	Mastectomy.
5 weeks before referral to our clinic	Radiotherapy for 25 days.
Day 0	The last fraction of radiation therapy.
Day 10	Symptoms that lasted for 10 days before referral to our clinic: dyspnea on light exertion and two-pillow orthopnea. Her symptoms were classified as New York Heart Association functional class III heart failure. Signs: Heart rate =110 bpm, respiratory rate =28 /min, and blood pressure =105/64 mmHg. On heart auscultation, she had S3 and S4, lung rales and crackle. A 12-lead electrocardiogram exhibited sinus tachycardia accompanied by nonspecific ST-T changes in the precordial leads. Premature ventricular count was 13.8% during 24-h Holter monitoring, accompanied by LV EF of 29% and global longitudinal strain of −11.2%.
Day 11	The results of serum tests showed a high sensitivity troponin I level 6 times above the normal limit (up to 14 ng/L), high levels of lactate dehydrogenase, creatinine phosphokinase, aspartate aminotransferase, C-reactive protein, and mild elevated total white blood cell count, as well as elevated NT pro-brain natriuretic peptide (6 times above the normal limit). Viral markers were negative.
Day 11	The results of cardiac magnetic resonance showed diffuse myocardial inflammation in T1 mapping, mid-wall late gadolinium enhancement in basal segments of interventricular septum, and subepicardial delayed enhancement in basal inferolateral segments.
Day 25	The patient was clinically stable without improvement of echocardiographic or STE findings (LV EF = 33%, global longitudinal strain =−11.9%).

LV EF, left ventricular ejection fraction; STE, speckle tracking echocardiography; GLS, global longitudinal strain.

**Table 2:** Transthoracic and speckle tracking echocardiographic findings: Baseline and postradiotherapy.

Dimensions	Baseline	Post-RT (day 10)
LVIDD (mm)	46	57
LVIDS (mm)	34	44
LVPW (D) (mm)	8	9
IVS (D) (mm)	8	9
LVPW (S) (mm)	10	10
IVS (S) (mm)	10	10
LVED volume (ml)	50.0	62
LVES Volume (ml)	24.8	44
LVEF (Simpson's) (%)	42	29
GLS (Average) (%)	−18.2	−11.2
RVIDD (Base) (mm)	33	32
TAPSE (mm)	13	13
IVC dimension (mm)	15	14
Left atrium (ES) (mm)	30	32
LAVI (ml/m^2^)	32	33
Sinus of Valsalva (mm)	31	31
AorticrRoot (mm)	29	30
Lateral e’ (cm/s)	8	7
Septal e’ (cm/s)	7	6
Mitral E/A	0.67	0.61

LVIDD, left ventricular internal dimension in diastole; LVIDS, left ventricular internal dimension in systole; LVPW, left ventricular posterior wall; LVED, left ventricular end diastole; LVES, left ventricular end systole; RVIDD, right ventricular internal dimension in diastole; TAPSE, tricuspid annular plane systolic excursion; IVC, inferior vena cava; ES, end systole; LAVI, left atrial volume index; IVS, interventricular septum; LV EF, left ventricular ejection fraction; GLS, global longitudinal strain.

## Discussion

Our case developed acute myocarditis after completing RT sessions. Pre-radiotherapy cardiac examinations, including ECG, echocardiography, and SPECT nuclear scan, were negative; therefore, the patient did not have any diagnosed coronary as well bypass graft failure in blood supply to the heart before radiation. Matching the postradiation clinical signs and results of cardiac examinations (reduced LV EF, reduced GLS, increased frequency of PVC complexes, and increased serum levels of inflammatory markers) with the European guideline of diagnosis of myocarditis guided the physician toward RIHD (10); diagnosis could be proved by biopsy analysis, but our patient refused to undergo cardiac biopsy. Therefore, CMR was performed for the patient, which showed acute myocarditis without an ischemic pattern. With the prescribed medications, the patient's clinical symptoms and signs improved and she had stable echocardiographic manifestations until the last follow-up.

Although the cardiotoxic effects of RT is a known quantity, the frequency and severity of myocardial damage are not parallel in all patients and depend on several factors, including the site of action, radiation dose, the method of administration, and patients’ characteristics, such as underlying CVDs or their risk factors, and the current or previous use of other antineoplastic therapies (11). Because of the proximity of the breast to the heart, RIHD can damage the heart and increase the risk of valvular heart disease, coronary artery disease, cardiomyopathy, congestive heart failure, and acute myocardial infarction, and pericarditis is higher in those with left-sided breast cancer (12, 13). Also, in the acute phase, most patients demonstrate ECG changes, such as abnormal heart rate, poor R wave progression and septal ST changes, RT-associated bundle branch block, and rarely complete heart block (14). The mechanism of RIHD is related to the microvascular ischemia that can disrupt the capillary endothelial framework (increase the capillary permeability) and cause hypoxia and injury to differentiated myocytes that result in the deposition of collagen and fibrosis (14, 15).

Patients with an underlying CVD and/or other diseases, including hypertension and DM, have also a higher vulnerability to the RT-induced endothelial damage and are thus at a higher risk of RIHD (11, 16); our patient also had a positive history of three-vessel disease and CABG 2 years before her mastectomy plus RT. The history of DM also predisposed the patient to endothelial dysfunction and systemic inflammatory reaction that could pave the path for the cardiotoxic effects of RT (16, 17). It is thus important to differentiate the RIHD from the cardiac diseases, such as cardiomyopathy or ischemic heart disease, the patient had before RT or developed after RT due to other causes. History of RT and the clinical signs and symptoms, suggested by the European guideline, can help with the diagnosis (10). It is worth mentioning that the combination of cancer treatment with chemotherapy has also been shown to increase the risk of RIHD in these patients (12, 18), but our patient had received no chemotherapy course at all.

Considering the high prevalence of breast cancer and the risk of damage to the adjacent organs, a high incidence has been reported for RIHD within 2 years of RT (11). Accordingly, several modifications have been suggested for sparing the heart from the toxic effects of RT, such as more accurate mapping by three-dimensional methods, additional maneuvers for displacing the heart from the radiation field, and advanced radiation methods (19), like intensity- and volumetric-modulated radiation therapy, to decrease the dose of radiation delivered to the heart, defined as the mean heart dose (MHD) (20). Also, the risk of RIHD has been shown to be positively associated with the radiation dose and increased per Gy of radiation (21). The cumulative risk of acute coronary event is reported to increase by 16.5% per Gy; the volume of the left ventricle receiving 5 Gy is identified as the most important prognostic dose–volume parameter (7). Despite the recommendations to reduce the MHD, some areas of the heart are exposed to higher radiation doses, which result in the occurrence of cardiac damage, even in small doses of radiation (16). 20–30 Gy has been considered as a high dose (6) and patients with a mediastinal radiation of ≤30 Gy had no increased risk for RIHD, while those >30 Gy reported a 3.5-fold increased risk. Of note, the radiotherapy dose in the case presented here did not pass 30 Gy (22). Thus, severe RIHD is not anticipated in this patient, although long-term follow-up should be performed for evaluating this issue. In addition to the above-mentioned factors, other patient-related risk factors, such as age at diagnosis, also influence the risk of RIHD development (7).

Pericardial fibrosis is caused by collagen deposition in the interstitium and parietal region of the thickened pericardium. Collagen deposition in the cardiac tissue results in RT-induced myocardial fibrosis that replaces cardiomyocytes (6). The oxidative injury and release of proinflammatory cytokines, altered coagulation and platelet activity, and altered permeability of cell membranes finally result in fibrosis and myocardial cell death (23, 24), which justifies the increased serum levels of inflammatory markers in the patient presented here. The cardiac biomarkers, hs Troponin I and NT pro-BNP, elevated in the patient presented here, have also been suggested as valuable serum biomarkers for early prediction of RIHD in patients with breast cancer (25, 26). Therefore, these markers have been suggested as available and easy methods to be used for the early screening of patients after RT (27). However, there is a lack of evidence-based literature for screening the presence and intensity of cardiac damage (28).

Abnormalities observed on ECG, such as arrhythmia, ST segment depression, and conduction block, can help diagnosis, but are not always present, and multimodality imaging techniques are suggested to be used for accurate diagnosis (29). Our patient also demonstrated an increased rate of PVCs on 24-h Holter ECG monitoring. Another issue concerning diagnosis is that the RT-induced myocardial fibrosis is mainly silent, and half of the cases that develop myocardial perfusion defects have no clinical signs and symptoms, sometimes found incidentally by TTE (11). In the case presented here, the patient had no clinical signs but showed reduced LV EF compared with that before mastectomy. However, our patient was a known case of CVD, and thus cardiac examination was performed for the patient, while diagnosis may be missed in patients with the late occurrence of cardiac damage, which includes most of the cases (30). One of the novelties of the case presented here is the occurrence of myocardial damage shortly after RT.

Although ECG and TTE are suggested as the preferred tools used for the assessment of the cardiovascular damage induced by RT, they may not be sensitive for the detection of myocardial fibrosis (27). Accordingly, CMR has been recommended for reflection of global and regional function, such as myocardial function and diagnosis of fibrosis (31). As STE has also been suggested as an appropriate tool for early diagnosis of RT-induced LV dysfunction (32), in the present study, we examined the myocardial strain using both CMR and STE, which helped the physician achieve the final diagnosis for the patient. The histopathological examination (and direct observation of myocardial cell degeneration) could have resulted in a definite diagnosis (31), not performed in our patient because of the lack of patient's consent.

The general therapeutic strategies suggested for RT-induced myocardial injury include antioxidants and anti-inflammatory agents, statins, and angiotensin-converting enzyme inhibitors; however, the treatment strategy is mainly selected individually based on the identified myocardial pathology and underlying diseases (33). In our case, the prescription of beta-blockers, mineralo recetore antagonist (MRA), furosemide, rosuvastatin, and low-dose oral steroid therapy resulted in clinical stability of the patient without changes in cardiac function (LV EF = 33%). Because of the lack of evidence on the best therapeutic approach in patients with acute myocarditis following RT, more studies are required to determine the best therapeutic approach for such patients.

## Conclusion

The case presented here suggested the necessity of detailed examination of patients after radiotherapy, not only for chronic occurrence of cardiomyopathy but also for the early occurrence of acute myocarditis. The three layers of the endocardium, myocardium, and epicardium can be affected by the RT, both at early and late phases. It has to be kept in mind that RIHD can also occur in the early phase, resulting in acute diseases, which can be of great significance. Therefore, it is suggested to pay greater attention to the risk factors of RIHD that can help the physician in the identification of patients with a higher risk. Also, the literature suggests that the available screening methods, such as serum tests, ECG, and TTE, can help with the diagnosis in some cases. However, the screening methods cannot result in a definite diagnosis. Here, we showed that STE and CMR could accurately diagnose LV dysfunction and myocarditis in a patient. However, further studies are required to determine the diagnostic accuracy of these two imaging methods compared with other imaging modalities in such patients in order to determine the best diagnostic tool for these patients. Also, the follow-up examination of the patient showed the appropriateness of the treatment strategy used for this patient, while there are no specific guidelines for the most appropriate treatment approach for such patients, and more studies are required in this regard.

## Data Availability

The raw data supporting the conclusions of this article will be made available by the authors, without undue reservation.

## Supplementary material

SUPPLEMENTARY FIGURE

Dose volume histogram of the radiation.
