# Supplementary figures and images for: Case report: Early acute myocarditis after radiation therapy for breast cancer: A case presentation and review of literature
Source: Front Cardiovasc Med. 2023 Apr 19;10:1020082. doi: 10.3389/fcvm.2023.1020082 (PMC10154576; doi:10.3389/fcvm.2023.1020082)

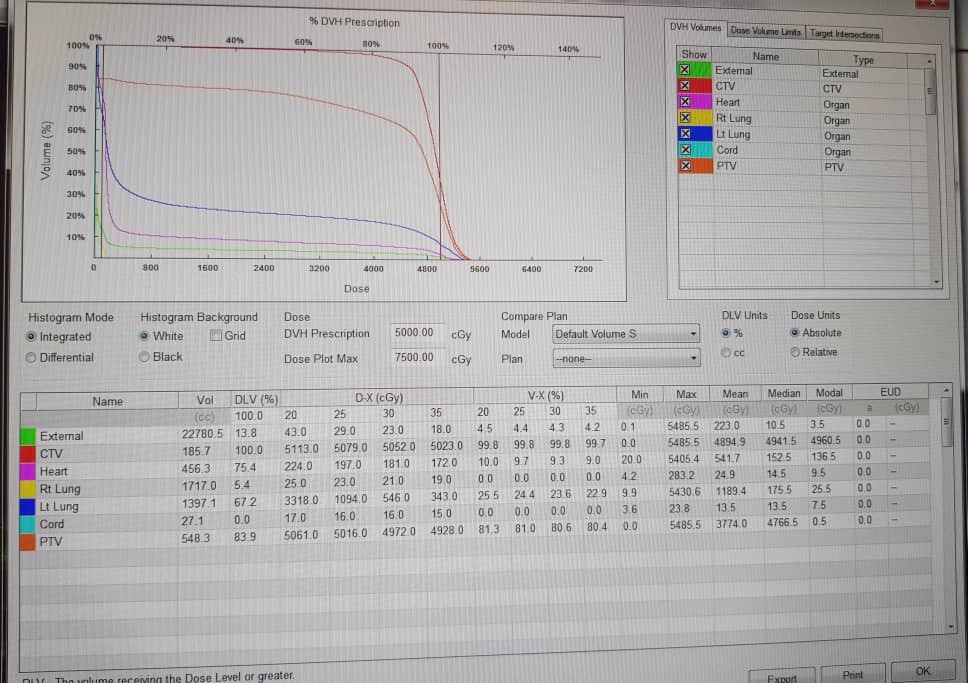

Supplement: Supplementary file 1 [file Image1.jpeg]
